# Discriminant Subspace Low-Rank Representation Algorithm for Electroencephalography-Based Alzheimer’s Disease Recognition

**DOI:** 10.3389/fnagi.2022.943436

**Published:** 2022-06-24

**Authors:** Tusheng Tang, Hui Li, Guohua Zhou, Xiaoqing Gu, Jing Xue

**Affiliations:** ^1^School of Computer Science and Information Engineering, Changzhou Institute of Technology, Changzhou, China; ^2^School of Information Engineering, Changzhou Institute of Industry Technology, Changzhou, China; ^3^School of Computer Science and Artificial Intelligence, Changzhou University, Changzhou, China; ^4^Department of Nephrology, The Affiliated Wuxi People’s Hospital of Nanjing Medical University, Wuxi, China

**Keywords:** electroencephalography, Alzheimer’s disease, low-rank representation, subspace learning, classification

## Abstract

Alzheimer’s disease (AD) is a chronic progressive neurodegenerative disease that often occurs in the elderly. Electroencephalography (EEG) signals have a strong correlation with neuropsychological test results and brain structural changes. It has become an effective aid in the early diagnosis of AD by exploiting abnormal brain activity. Because the original EEG has the characteristics of weak amplitude, strong background noise and randomness, the research on intelligent AD recognition based on machine learning is still in the exploratory stage. This paper proposes the discriminant subspace low-rank representation (DSLRR) algorithm for EEG-based AD and mild cognitive impairment (MCI) recognition. The subspace learning and low-rank representation are flexibly integrated into a feature representation model. On the one hand, based on the low-rank representation, the graph discriminant embedding is introduced to constrain the representation coefficients, so that the robust representation coefficients can preserve the local manifold structure of the EEG data. On the other hand, the least squares regression, principle component analysis, and global graph embedding are introduced into the subspace learning, to make the model more discriminative. The objective function of DSLRR is solved by the inexact augmented Lagrange multiplier method. The experimental results show that the DSLRR algorithm has good classification performance, which is helpful for in-depth research on AD and MCI recognition.

## Introduction

Alzheimer’s disease (AD) is a disease characterized by memory loss, slow and gradual changes in brain function, and the manifestations of intellectual loss (Zhang et al., 2021). With the advancement of global aging, AD has now become a major public health problem affecting the world. The existing treatment of AD can only temporarily help relieve memory and cognition, but not a cure. To obtain disease-controlling treatments, it is an urgent need to classify the course of AD for early diagnosis. And especially, the National Institutes of Health revised the clinical diagnostic criteria for AD, characterizing research guidelines for early diagnosis and treatment (Cummings, 2021). The progression of AD is mainly divided into three stages. The first is the early clinical stage with no symptoms; the second is the intermediate stage with mild cognitive impairment (MCI); and the final stage with dementia symptoms (Mirzaei and Adeli, 2022).

More researchers are studying methods that can sensitively and conveniently monitor AD, involving cognitive neuropsychological detection, biochemical detection, neuroimaging detection, and so on. In recent years, electroencephalography (EEG) has become an important tool for studying human brain activity (Ghorbanian et al., 2015). Noninvasive EEG imaging methods are directly related to neural local field potentials and have a high temporal resolution. The millisecond-level temporal resolution and direct electrophysiological information provided by EEG can accurately reflect cognitive behaviors related to human neural activity. Therefore, more studies are beginning to use EEG for the diagnosis and prediction of early AD. For example, EEG spectral studies have revealed that EEG diffuse slow waves are a major feature of AD. EEG studies of AD patients have shown that the reduced power in the alpha (8–15 Hz) band and the increased power in the delta (0.5–4 Hz) band are significant features of AD (Fröhlich et al., 2021). The increase in power in the theta (4–8 Hz) band and the decrease in power in the beta (15–30 Hz) band also indicate that they can be useful for detecting MCI to AD transitions (Maturana-Candelas et al., 2020). Recently, machine learning technology has been widely used in the analysis of brain imaging data, which has greatly promoted the development of cognitive neuroscience. Most of the research revolves around feature extraction and classifier optimization. In terms of feature extraction, Wen et al. (2020) first converted the EEG signals into multispectral images and then used a deep convolutional neural network learning model for EEG classification. Similarly, Ieracitano et al. (2019a) drew the power spectral density of the EEG into the form of a spectrogram, and converted the EEG signal classification into a CNN-based image classification problem. Ieracitano et al. (2019b) spliced the continuous wavelet transform features and bispectral features of EEG signals to achieve the fusion of the two types of features. The advantage of this algorithm is that the fused features can obtain higher accuracy than only using one type of feature. The disadvantage is that the correlation between features is not considered enough. At the same time, the dimension of fusion features is greatly increased, which is easy causing the over-fitting problem.

In terms of classification algorithms, Miltiadous et al. (2021) compared six classification algorithms for EEG analysis for frontotemporal dementia in AD and verified the effectiveness of these algorithms. This study provided solutions for the early diagnosis of frontotemporal dementia. Anuradha and Jamal (2021) detected the progression of AD by detecting abnormal behavior in EEG. The authors used a feed-forward artificial neural network as a classifier to perform EEG feature analysis on abnormal and normal subjects and obtained a classification accuracy of 94.4%. Ge et al. (2020) exploited the robust biomarkers in EEG, combined linear discriminant analysis as a classifier, and proposed a systematic identification framework based on signal processing and computer-aided techniques for the detection of AD. Araujo et al. (2022) developed an intelligent system that can distinguish various stages of AD through EEG signals. The system used wavelet packet to extract multi-band features of EEG signals and used multiple machine learning methods as classification models.

Electroencephalography signals can reflect the functional state of the brain and the activity of brain physiological structures. The difficulties in classifying EEG signals using machine learning algorithms are as follows: first, the amplitude of the EEG signals is usually around 50 μv. The EEG signals are very weak, and their background noise is usually very strong. Second, EEG signals have strong randomness. In the process of acquisition, EEG signals will not only be stimulated by the outside world but also produce interference signals due to their own blinking and other actions. Therefore, it is still a challenging task to use machine learning methods to identify AD based on EEG signals. To solve this problem, the researchers usually reduce the dimension of EEG high-dimensional data and extract a small amount of the most valuable compact information, which not only saves storage space and processing time but also enables learning a robust model (Lei et al., 2021). Subspace learning and low-rank representation can well achieve this goal. Subspace learning is a well-known dimension reduction method in machine learning. Its main goal is to adopt appropriate strategies to map high-dimensional original data into the low-dimensional subspace to reduce the data dimension. Low-rank representation (LRR) can effectively separate the noise in the EEG signals to restore clean data and obtain accurate subspace segmentation of data.

Inspired by the strong theory of subspace learning and low-rank representations, this paper proposes an EEG-based discriminant subspace low-rank representation learning algorithm (DSLRR) for AD recognition. On the one hand, based on the low-rank representation, DSLRR utilizes the supervised information and local manifold information by least squares regression (LSR) and graph discriminant embedding. On the other hand, DSLRR introduces principal component analysis (PCA) and global preserved constraints into the subspace of learning. The algorithm optimization adopts a strategy of alternating parameter updates using the inexact augmented Lagrange multiplier method. Our contribution is as follows: (1) The DSLRR algorithm combines subspace learning and low-rank representation in a flexible manner. (2) By introducing global graph embedding and PCA term, the data projection can preserve the global structure information of EEG data in the discriminant subspace. (3) The learned low-rank representation coefficient can effectively avoid the negative effects of the original data’s redundant features and noise information. (4) By introducing LSR and graph discriminant embedding, the learned low-rank representation coefficient can explicitly contain the intrinsic local manifold structure and discriminant information of EEG data. The experiments on four EEG datasets verify that the DSLRR algorithm can be effectively used for the recognition of AD, MCI, and healthy control (HC).

## Background

### Electroencephalography Dataset for Alzheimer’s Disease and Mild Cognitive Impairment Recognition

**FIGURE 1:**
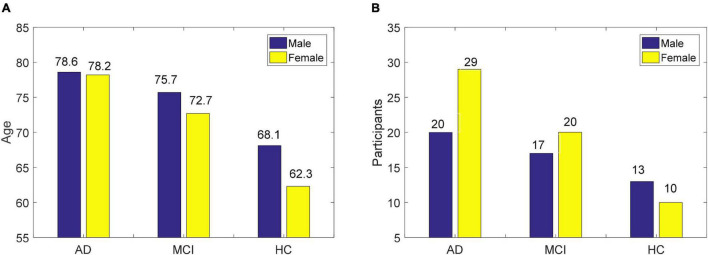
The basic information of EEG data used in this study, **(A)** age of men and women, and **(B)** proportion of gender.

The EEG data were obtained from 109 participants recruited at the IRCCS Centro Neurolesi Bonino-Pulejo in Italy, including 23 HC, 49 AD, and 37 MCI (Fiscon et al., 2018). The age of men and women and the proportion of genders are shown in Figure 1. The EEG data collection time was from 2012 to 2013. The scalp electrode position was determined using the international 10–20 system, and EEG data from 19 electrodes were collected. The sampling frequency was 256 or 1,024, and the acquisition time of EEG signals was 300 s. To reduce the effect of the artifact, the EEG signals from 60 to 240 s were selected, and the adopted normalized sampling frequency was 256 Hz. Feature extraction adopted the fast Fourier transform, which divided 180 s of data into six epochs of 30 s, and extracted 16 Fourier coefficients. Therefore, 304 features (19 electrodes × 16 Fourier coefficients) were available for each sample.

### Subspace Learning

We have a labeled dataset with *n* samples *Y* = [*y*_1_,…,*y*_*n*_] ∈ *R*^*d*×*n*^, where *y*_*i*_ represents the *i*th training sample, and its class label matrix is $\bar{Y}=\left[{\bar{y}}_{1},\ldots ,{\bar{y}}_{n}\right]\in R^{C\times n}$. The dimension of the sample is *d*, and *n* samples are divided into *C* classes. When the dimensionality of the original EEG data is high, the data computational and storage costs will be very large. Thus, a common solution is to project the high-dimensional data into a low-dimensional space (Lei et al., 2021). Let *Q* ∈ *R*^*d*×*C*^ be the projection matrix, the projection data can be represented as *V* = [*v*_1_,…,*v*_*n*_] ∈ *R*^*C*×*n*^ in the label space, where *V* = *Q^T^Y*.

Generally speaking, the premise of manifold subspace learning is that the data exists in high-dimensional space in the form of manifold embedding from low-dimensional space data. The key point of manifold learning is to ensure that low-dimensional data can reflect the inherent structural information contained in high-dimensional space (Zhang et al., 2020). As a commonly used manifold learning algorithm, locality-preserving projection (LPP) preserves the local neighbor relationship of the data by using an adjacency graph and affinity matrix (Weng and Shen, 2008). The LPP algorithm consists of three steps. Step 1 is to construct an adjacency graph. For example, we construct an adjacency graph using the *k*-nearest neighbor algorithm. The nearest neighbors of each point connected to it are known as neighbor nodes. Step 2 is to assign weights to each edge. In the adjacency graph, the affinity matrix represents the similarity between sample points, which can generally be calculated using the two-value method, cosine distance or Gaussian kernel function. For example, the affinity matrix **E** constructed by the two-value method can be defined as follows:


(1) $$E_{\text{ij}}=\left\{ \begin{array}{c} 1,\text{if}\,y_{i}\in N_{k}\,\left(y_{j}\right)\,\text{or}\,y_{j}\in N_{k}\,\left(y_{i}\right)\\ 0,\text{otherwise} \end{array}\right.$$

where *N*_*k*_(*y*_*i*_) represents the *k* nearest neighbor nodes of *y*_*i*_.

### Low-Rank Representation

Low-rank representation aims to exploit the sparsity of matrix singular values to model high-dimensional data in multi subspace (Li et al., 2017; Jiang et al., 2021). Given a dataset **Y**, the LRR algorithm regards the input data itself as a dictionary and uses the basis in the dictionary to linearly represent the sample points, while minimizing its rank. The optimization problem of LRR can be described as follows:


(2) $$\begin{array}{c} \min_{L}\mathrm{rank}\,\left(L\right),\\ s.t.Y=Y\,L, \end{array}$$

where *L* ∈ *R*^*n*×*n*^ is the representation coefficients of **Y**, which reflects the global correlation between the original data samples. In theory, the coefficient matrix **L** obtained by the LRR should be a block diagonal matrix. That is to say, each block corresponds to a subspace, the number of blocks represents the number of data subspaces, and the size of the block corresponds to the dimension of the subspace.

Eq. (2) is not a convex optimization problem due to its discrete. Using the nuclear norm instead of *rank*(*L*), Eq. (2) can be transformed into the convex optimization problem as:


(3) $$\begin{array}{c} \min_{L}{||L||}_{*},\\ s.t.Y=Y\,L, \end{array}$$

where ||||_*_ is the nuclear norm.

Considering the noise or sparse error in **Y**, LRR enhances the model’s robustness by improving the correlation between the individual columns of **L**, and the problem of LRR can be written as:


(4) $$\begin{array}{c} \min_{L,S}{||L||}_{*}+\theta \,{||S||}_{1},\\ s.t.Y=Y\,L+S, \end{array}$$

where *S* ∈ *R*^*d*×*n*^ is sparse component of **Y**. θ is the regularization parameter.

Obviously, LRR decomposes the data **Y** into low-rank representation *YL* and sparse representation **S**. The former component *YL* generally represents the main features contained in **Y**, and the latter generally represents the redundant features and noise information contained in **Y**. In the clean data scenario, *S* represents the reconstruction error. Therefore, **L** can accurately indicate the subspace segmentation of **Y**, which ensures the robustness of the learned model. However, LRR ignores the role of local structure information in data and does not exploit the supervised information in the training data. Therefore, LRR cannot reflect the intra-class identity and inter-class dissimilarity in low-rank representation.

## Discriminant Subspace Low-Rank Representation Algorithm

### Objective Function

#### Discriminant Margin Term on Representation Coefficients

To learn the discriminant low-rank representations, we introduce graph discriminant embedding (Huang et al., 2018) into our algorithm, which combines supervised information to define intra-class and inter-class graph affinity matrices. We think if two EEG samples are closer in the original space, their representation coefficients will be close to each other. The compactness between samples of the same class and the separability between samples of different classes is the important knowledge in discriminant low-rank representations. To this end, we define affinity matrices *E*^com^ and *E*^sep^ to represent the similar relationship between intra-class and inter-class, respectively:


(5) $$E_{i,j}^{\text{com}}=\left\{ \begin{array}{c} e\,\frac{-{||y_{i}-y_{j}||}^{2}}{t},\text{if}\,y_{i}\in {\hat{N}}_{k}\,\left(y_{i}\right)\,\text{or}\,y_{j}\in {\hat{N}}_{k}\,\left(y_{i}\right),{\bar{y}}_{j}={\bar{y}}_{i}\\ e\,\frac{-t}{{||y_{i}-y_{j}||}^{2}},\text{if}\,y_{i}\notin {\hat{N}}_{k}\,\left(y_{i}\right)\,\text{or}\,y_{j}\notin {\hat{N}}_{k}\,\left(y_{i}\right),{\bar{y}}_{j}={\bar{y}}_{i}\\ 0,\text{if}\,{\bar{y}}_{j}\neq {\bar{y}}_{i} \end{array}\right.$$

(6) $$E_{i,j}^{\text{sep}}=\left\{ \begin{array}{c} e\,\frac{-{||y_{i}-y_{j}||}^{2}}{t},\text{if}\,y_{i}\in {\tilde{N}}_{k}\,\left(y_{i}\right)\,\text{or}\,y_{j}\in {\tilde{N}}_{k}\,\left(y_{i}\right),{\bar{y}}_{j}\neq {\bar{y}}_{i}\\ e\,\frac{-t}{{||y_{i}-y_{j}||}^{2}},i\,\text{f}\,y_{i}\notin {\tilde{N}}_{k}\,\left(y_{i}\right)\,\text{or}\,y_{j}\notin {\tilde{N}}_{k}\,\left(y_{i}\right),{\bar{y}}_{j}\neq {\bar{y}}_{i}\\ 0,\text{if}\,{\bar{y}}_{j}={\bar{y}}_{i} \end{array}\right.$$

where ${\hat{N}}_{k}\,\left(\right)$ and ${\tilde{N}}_{k}\,\left(\right)$ represent the *k*-nearest neighbor samples of intra-class and inter-class, respectively. The parameter *t* (*t* > 0) is the weight parameter used to adjust the correlation between two samples. We set *t* = 1 in this study.

Then we define the discriminant margin term ς_1_(*L*) on representation coefficients:


(7) $$\begin{array}{c} \varsigma_{1}\,\left(L\right)=\sum_{i=1}^{n}\varsigma_{i}\,\left(L_{i}\right)\\ =\sum_{i=1}^{n}\sum_{j=1}^{n}\left({||L_{i}-L_{j}||}^{2}\,E_{i,j}^{\text{com}}-{||L_{i}-L_{j}||}^{2}\,E_{i,j}^{\text{sep}}\right)\\ =\text{Tr}\,\left(L^{T}\,U\,L\right) \end{array}$$

where *U* = *E*^com^−*E*^sep^ + ε*I*, ε is a very small positive. Eq. (7) represents the intra-class compactness and the inter-class dissimilarity in representation coefficients. Its essence is to excavate the local structural information representation coefficients. In addition, Eq. (7) can avoid the influence of the redundant information and noise of the original data.

#### Global Structure Term on Projection

We adopt the affinity matrix **E** to represent the correlation between two samples using supervised information. The element *e*_*ij*_ in **E** is computed as:


(8) $$e_{i\,j}=\left\{ \begin{array}{c} 1,\text{if}\,y_{i}\,\text{and}\,y_{j}\,\text{are}\,\text{of}\,\text{the}\,\text{same}\,\text{class}\\ 0,\text{otherwise} \end{array}\right.$$

To preserve the global discriminant information of the original data in the subspace, we introduce the global structure term on projection:


(9) $$\begin{array}{c} \varsigma_{2}\,\left(Q\right)=\frac{1}{2}\,\sum_{i,j}e_{\text{ij}}\,{||Q^{T}\,y_{i}-Q^{T}\,y_{j}||}_{2}^{2}-\beta \,\text{Tr}\,\left(Q^{T}\,Y\,Y^{T}\,Q\right)\\ =\text{Tr}\,\left(Q^{T}\,Y\,E\,Y^{T}\,Q\right)-\beta \,\text{Tr}\,\left(Q^{T}\,Y\,Y^{T}\,Q\right)\\ =\text{Tr}\,\left(Q^{T}\,Y\,\left(E-\beta \,I\right)\,Y^{T}\,Q\right) \end{array}$$

where β is the regularization parameter.

The first factor $\sum_{i,j}e_{i\,j}\,{||Q^{T}\,y_{i}-Q^{T}\,y_{j}||}_{2}^{2}$ in Eq. (9) is the global preserved component on projection. Obviously, when this component reaches the minimum, the distance of samples of the same class will be as close as possible in the projection subspace. The second component Tr(*Q^T^YY^T^Q*) in Eq. (9) is the PCA component on projection. Its goal is to ensure that the projecting data in the low-dimensional subspace can depict the inherent structure information contained in the original space.

#### Least Squares Regression Term

As an effective supervised learning method, LSR learns the linear projection that transforms the sample to the label space, and obtains the regression vector as the data representation in the label space (Zhao et al., 2022). Therefore, we try to find a projection matrix with the help of LSR in the low-rank representation. Different from the traditional projection method on the original data, the DSLRR algorithm only uses clean data representation to learn the projection matrix in the low-rank representation framework, which can not be affected by the redundant information of EEG data. This idea can be obtained as:


(10) $$\begin{array}{c} \varsigma_{3}\,\left(Q,L,S\right)={||L||}_{*}+\theta \,{||S||}_{1}+\gamma \,{||V-\bar{Y}||}_{F}^{2}+\eta \,{||V||}_{F}^{2},\\ s.t.Y=Y\,L+S,\\ V=Q^{T}\,Y\,L,\\ 1_{n}^{T}\,L=1_{n}^{T}. \end{array}$$

where γ and η are regularization parameters.

Equation (10) tries to minimize the least squares loss between the regression results and the corresponding regression target. In addition, in the low-rank representation framework, the compact representation of the data can be learned through subspace projection.

#### The Objective Function

We integrate Eqs (7), (9), and (10) into a learning model, and obtain the objective function of the DSLRR algorithm:


(11) $$\begin{array}{c} \min\varsigma_{1}\,\left(L\right)+\varsigma_{2}\,\left(Q\right)+\varsigma_{3}\,\left(Q,L,S\right)\\ =\min_{Q,L,S}\mu \,\text{Tr}\,\left(L^{T}\,U\,L\right)+\alpha \,\text{Tr}\,\left(Q^{T}\,Y\,\left(E-\beta \,I\right)\,Y^{T}\,Q\right)+{||L||}_{*}+\\ \theta \,{||S||}_{1}+\gamma \,{||V-\bar{Y}||}_{F}^{2}+\eta \,{||V||}_{F}^{2},\\ s.t.Y=Y\,L+S,\\ V=Q^{T}\,Y\,L,\\ 1_{n}^{T}\,L=1_{n}^{T}. \end{array}$$

where α and μ are regularization parameters.

From Eq. (11), we can see that the DSLRR algorithm combines subspace learning and low-rank representation into a learning model. Based on low-rank representation learning, the compact and discriminant low-rank representation can be reinforced by graph discriminant embedding. Based on subspace learning, the discriminant projection can be obtained by LSR, global structure preserved, and PCA technologies.

### Optimization

There are three unsolved parameters {**Q**, **L**, **S**} in Eq. (11). To make Eq. (11) separable, the relaxation matrix Λ is introduced to represent *L*. Substitute the constraint *V* = *Q^T^YL* into Eq. (11), Eq. (11) can be re-written as:


(12) $$\begin{array}{c} \min_{Q,L,S}{||\Lambda ||}_{*}+\theta \,{||S||}_{1}+\gamma \,{||Q^{T}\,Y\,L-\bar{Y}||}_{F}^{2}+\eta \,{||Q^{T}\,Y\,L||}_{F}^{2}+\\ \mu \,\text{Tr}\,\left(L^{T}\,U\,L\right)+\alpha \,\text{Tr}\,\left(Q^{T}\,Y\,\left(E-\beta \,I\right)\,Y^{T}\,Q\right),\\ s.t.Y=Y\,L+S,\\ 1_{n}^{T}\,L=1_{n}^{T},\\ L=\Lambda . \end{array}$$

We optimize three parameters by the inexact augmented Lagrange multiplier algorithm in an iterative optimization strategy (Kang et al., 2015). Eq. (12) has the following form:


(13) $$\begin{array}{c} \min_{Q,L,S,\Lambda }{||\Lambda ||}_{*}+\theta \,{||S||}_{1}+\gamma \,{||Q^{T}\,Y\,L-\bar{Y}||}_{F}^{2}+\eta \,{||Q^{T}\,Y\,L||}_{F}^{2}+\\ \mu \,\text{Tr}\,\left(L^{T}\,U\,L\right)+\alpha \,\text{Tr}\,\left(Q^{T}\,Y\,\left(E-\beta \,I\right)\,Y^{T}\,Q\right)+\text{Tr}\,\left(\tau_{a}^{T}\,\left(Y-Y\,L-S\right)\right)\\ +\text{Tr}\left(\tau_{b}^{T}\left(\Lambda -L\right)\right)+\text{Tr}\left(\tau_{c}^{T}\left(1_{n}^{T}L-1_{n}^{T}\right)\right)+\frac{\delta }{2}(||\text{Y-YL}-S|{|}_{F}^{2}+\\ ||\Lambda -L|{|}_{F}^{2}+||1_{n}^{T}L-1_{n}^{T}|{|}_{F}^{2}), \end{array}$$

where δ is a trade-off parameter. The matrices τ_*a*_ ∈ *R*^*d*×*n*^, τ_*b*_ ∈ *R*^*d*×*n*^, τ_*c*_ ∈ *R*^*d*×*n*^, and τ_*d*_ ∈ *R*^*d*×*n*^ are the Lagrange multipliers.

1) Optimize **Q**, while fixing the other parameters. Eq. (13) can be written as:


(14) $$\min_{Q}\gamma \,{||Q^{T}\,Y\,L-\bar{Y}||}_{F}^{2}+\eta \,{||Q^{T}\,Y\,L||}_{F}^{2}+\alpha \,\text{Tr}\,\left(Q^{T}\,Y\,\left(E-\beta \,I\right)\,Y^{T}\,Q\right).$$

We can get the closed-solution of **Q** as:


(15) $$Q={\left[Y\,\left(\left(\alpha \,\left(E-\beta \,I\right)\right)+\left(\gamma +\eta \right)\,L\,L^{T}\right)\,Y^{T}\right]}^{-1}\,Y\,L\,{\bar{Y}}^{T}.$$

2) Optimize Λ, while fixing the other parameters. Eq. (13) can be written as:


(16) $$\begin{array}{c} \min_{\Lambda }{||\Lambda ||}_{*}+\text{Tr}\,\left(\tau_{c}^{T}\,\left(L-\Lambda \right)\right)+\frac{\delta }{2}\,{||L-\Lambda ||}_{F}^{2}\\ =\min_{\Lambda }\frac{1}{\delta }\,{||\Lambda ||}_{*}+\frac{1}{2}\,{||\Lambda -\left(L+\frac{1}{\delta }\,\tau_{c}\right)||}_{F}^{2}. \end{array}$$

We use the singular value thresholding operator (Cai et al., 2010; Li et al., 2017) to solve Eq. (16). We employ the singular value decomposition algorithm on $L+\frac{1}{\delta }\,\tau_{c}$ as $L+\frac{1}{\delta }\,\tau_{c}=\text{H}\,\Sigma \,\Delta$, where H is the diagonal matrix with its element being a group of singular values {Θ_*k*_},1≤*k*≤*p*, *p* is the rank. The matrix Λ can be computed by Λ = HΩ_(1/δ)_ΣΔ, in which $\Omega_{\left(1/\delta \right)}\,\Sigma =d\,i\,a\,g\,\left({\left\{\Theta_{k}-\frac{1}{\delta }\right\}}_{+}\right)$, where “+” means the positive part.

3) Optimize **L**, while fixing the other parameters. Eq. (13) can be written as:


(17) $$\begin{array}{c} \min_{L}\gamma \,{||Q^{T}\,Y\,L-\bar{Y}||}_{F}^{2}+\eta \,{||Q^{T}\,Y\,L||}_{F}^{2}+\mu \,\text{Tr}\,\left(L^{T}\,U\,L\right)+\\ \text{Tr}\,\left(\tau_{a}^{T}\,\left(Y-Y\,L-S\right)\right)+\text{Tr}\,\left(\tau_{b}^{T}\,\left(\Lambda -L\right)\right)+\text{Tr}\,\left(\tau_{c}^{T}\,\left(1_{n}^{T}\,L-1_{n}^{T}\right)\right)+\\ \frac{\delta }{2}\,\left({||\text{Y-YL}-S||}_{F}^{2}+{||\Lambda -L||}_{F}^{2}+{||1_{n}^{T}\,L-1_{n}^{T}||}_{F}^{2}\right), \end{array}$$

Let the first derivative of **L** in Eq. (16) be zero, we have,


(18) $$\Theta_{a}\,L=\Theta_{b}+\Theta_{c},$$

(19) $$\Theta_{a}=2\,U+2\,\left(\gamma +\eta \right)\,Y^{T}\,Q\,Q^{T}\,Y+\delta \,\left(Y^{T}\,Y+1_{n}\,1_{n}^{T}+I_{n}\right),$$

(20) $$\Theta_{b}=2\,\gamma \,Y^{T}\,Q\,\bar{Y}+\delta \,\left(Y^{T}\,{\text{Y-Y}}^{T}\,S+\Lambda +1_{n}\,1_{n}^{T}\right),$$

(21) $$\Theta_{c}=Y^{T}\,\tau_{a}-\tau_{b}-1_{n}\,\tau_{c},$$

We can get the closed-solution of **L** as:


(22) $$L=\Theta_{a}^{-1}\,\left(\Theta_{b}+\Theta_{c}\right).$$

4) Optimize **S**, while fixing the other parameters. Eq. (13) can be written as:


(23) $$\min_{S}\frac{\theta }{\delta }\,{||S||}_{2,1}+\frac{1}{2}\,{||S-\left(Y-Y\,L+\frac{1}{\delta }\,\tau_{a}\right)||}_{F}^{2},$$

According to the theory of (Liu et al., 2013), we can obtain the **S** by


(24) $$S\,\left(:,i\right)=\left\{ \begin{array}{c} \frac{||\tau_{i}||-\theta }{||\tau_{i}||}\,||\tau_{i}||,i\,f\,\frac{\theta }{\delta }<||\tau_{i}||\\ 0,\text{otherwise} \end{array}\right.$$

where τ_*i*_ is the *i*th column vector of the matrix τ_*a*_.

### Testing

Given test EEG data *Y*_test_, we first compute its low-rank representation *L*_test_ using Eq. (11), while setting parameters γ = 0, α = 0, and μ = 0. Second, we construct the new training set *YL* and test set *Y*_test_*L*_test_. Third, we use the training set *YL* to train a classifier and build a classifier to predict the label of *Y*_test_*L*_test_. In this study, we used nearest neighbor (NN) algorithm as the classifier. The whole training and testing procedure for EEG data recognition are summarized in Algorithm 1.

**ALGORITHM 1:** DSLRR algorithm for EEG data recognition.

Input: The training EEG data *Y* and its label $\bar{Y}$, the testing EEG data *Y*_test_;
Output: the class label of *Y*_test_;
// Construct the new training data on *Y*
Calculate matrices E by Eq. (8), *E*^com^ by Eq. (5) and *E*^sep^ by Eq. (6);
Repeat:
Optimize Q using Eq. (15) withΛ, L, and S fixed;
OptimizeΛ using Eq. (16) with Q, L, and S fixed;
Optimize L using Eq. (22) withΛ, Q, and S fixed;
Optimize S using Eq. (24) withΛ, L, and Q fixed;
Obtain the new testing data *YL*;
Until Eq. (13) convergence
// Construct the new testing data on *Y*_test_
Repeat:
Optimize Q using Eq. (15) withΛ, L, and S fixed, while setting γ = 0, α = 0, and μ = 0;
OptimizeΛ using Eq. (16) with Q, L, and S fixed, while setting γ = 0, α = 0, and μ = 0;
Optimize L using Eq. (22) withΛ, Q, and S fixed, while setting γ = 0, α = 0, and μ = 0;
Optimize S using Eq. (24) withΛ, L, and Q fixed, while setting γ = 0, α = 0, and μ = 0;
Until Eq. (13) convergence
Obtain the new test data *Y*_test_*L*_test_;
// Train a classifier and predict the class label
Train a classifier using training data *YL* (such as NN classifier, support vector machine);
Test and output the class label of *Y*_test_*L*_test_ using the trained classifier.

## Experiment

### Experimental Settings

To verify the effectiveness of the DSLRR algorithm, we compared the DSLRR algorithm with the SPCA (Jiang, 2011), LRR (Liu et al., 2013), LRDLSR (Chen and Yang, 2014), JSLC (Lu et al., 2021), and NRLRL (Gao et al., 2021) in the experiment. The LRR algorithm is the baseline algorithm of the DSLRR algorithm. SPCA and JSLC algorithms are subspace learning algorithms. LRDLSR and NRLRL are low-rank representation algorithms. For SPCA, the weight parameters are set in the covariance mixture, α is set inversely proportional to the sample size, and η is searched in [2^−5^,2^−4^,…,2^5^]. For LRR, the parameter λ is searched in $\left[1,4,\ldots ,30\right]/\sqrt{d}$, where *d* is the data dimension. For LRDLSR, the parameters α and β are searched in [10^−4^,10^−3^,…,1], and the parameters γ and λ are set to be 0.01. For JSLC, subspace dimension and the size of the dictionary are searched in [50, 100,…, 300]. The regularization parameters are searched in [0.5, 1,…, 5]. For NRLRL, the size of the dictionary is searched in [50, 100,…, 300], and λ, γ, and η are searched in [2^−4^,2^−3^,…,2^4^]. For DSLRR, all regularization parameters are searched in[2^−4^,2^−3^,…,2^4^], and *k*-nearest neighbors in ${\hat{N}}_{k}\,\left(\right)$ and ${\tilde{N}}_{k}\,\left(\right)$ are searched in [1,…, 11].

Due to the limited training EEG samples, we expand the EEG data with the data augmentation strategy. The number of EEG samples in HC, MCI, and AD is 69, 74, and 98, respectively. In this section, the experiments are conducted on four EEG datasets for AD and MCI recognition, namely, (1) HC & AD, (2) HC & MCI, (3) HC & (MCI+AD), and (4) MCI & AD. The ratio of the two classes of samples is 1:1. We randomly select 50 samples in each class for model training, and the rest samples are used for testing. We perform our experiments 10 times and record the classification performance in terms of accuracy, sensitivity, specificity, precision, F-measure, G-mean, and Jaccard. All experiments are conducted by MATLAB on a Windows machine.

### Classification Results

The classification results in four EEG datasets are reported in Tables 1–4, where the best results are highlighted in bold. These four data sets are binary classification problems. According to the results in Tables 1–4, we can see that:

**TABLE 1:** Classification results of the comparison algorithms in HC and AD dataset.

	Accuracy	Sensitivity	Specificity	Precision	F-measure	G-mean	Jaccard
SPCA	92.54	92.11	92.98	93.51	92.46	92.35	86.33
	±2.68	±1.58	±2.09	±2.22	±2.69	±2.75	±3.03
LRR	88.16	92.11	84.21	85.29	88.45	87.99	79.55
	±2.56	±2.06	±1.53	±2.24	±1.79	±1.53	±2.51
LRDLSR	93.42	**99.87**	86.84	88.42	93.84	93.18	88.42
	±2.78	±2.03	±2.72	±2.86	±2.78	±2.77	±2.78
JSLC	94.68	96.84	92.63	93.10	94.87	94.66	90.82
	±1.93	±1.66	±1.80	±2.59	±3.55	±2.01	±1.49
NRLRL	96.05	92.11	99.70	99.74	95.71	95.88	92.11
	±3.03	±1.08	±1.36	±2.08	±2.99	±2.78	±3.46
Our algorithm	**97.74**	95.49	**99.81**	**99.79**	**97.55**	**97.65**	**95.49**
	±2.68	±2.14	±1.57	±1.91	±3.19	±1.64	±1.72

*The bold values mean the best performance results.*

**TABLE 2:** Classification results of the comparison algorithms in MCI and AD dataset.

	Accuracy	Sensitivity	Specificity	Precision	F-measure	G-mean	Jaccard
SPCA	91.12	91.71	90.53	91.71	91.01	90.70	84.19
	±3.23	±3.24	±2.62	±2.92	±3.00	±1.77	±2.54
LRR	87.89	80.42	95.37	94.77	86.23	87.15	76.85
	±1.99	±3.20	±2.39	±2.95	±2.61	±1.79	±2.52
LRDLSR	92.32	84.32	**99.89**	**99.90**	90.46	91.93	84.32
	±1.39	±2.09	±2.98	±2.78	±2.98	±2.91	±2.55
JSLC	92.98	88.42	97.54	97.70	92.04	92.46	86.41
	±2.49	±2.38	±2.44	±2.55	±2.60	±2.08	±1.78
NRLRL	94.74	89.47	99.53	99.47	94.29	94.51	89.47
	±2.13	±3.34	±2.33	±1.69	±2.22	±3.29	±3.88
Our algorithm	**95.61**	**94.74**	96.49	96.67	**95.43**	**95.48**	**91.40**
	±1.83	±2.09	±1.75	±2.35	±2.19	±2.04	±2.67

*The bold values mean the best performance results.*

**TABLE 3:** Classification results of the comparison algorithms in HC and (MCI+AD) dataset.

	Accuracy	Sensitivity	Specificity	Precision	F-measure	G-mean	Jaccard
SPCA	92.11	84.21	99.25	99.13	91.43	91.77	84.21
	±2.62	±1.39	±2.98	±2.53	±2.37	±2.46	±2.12
LRR	89.47	84.21	94.74	94.12	88.89	89.32	80.00
	±3.48	±3.08	±1.94	±3.43	±2.92	±2.34	±2.28
LRDLSR	94.74	94.74	94.74	95.16	94.62	94.56	89.90
	±3.08	±3.17	±3.63	±1.25	±2.38	±2.44	±1.37
JSLC	95.61	94.74	96.49	96.37	95.44	95.55	91.67
	±2.18	±1.91	±2.60	±2.46	±1.57	±1.61	±2.67
NRLRL	96.26	92.22	**99.43**	**99.35**	95.86	95.99	92.26
	±2.78	±3.57	±1.62	±3.38	±3.78	±1.89	±3.38
Our algorithm	**98.42**	**99.34**	96.84	97.00	**98.46**	**98.40**	**97.00**
	±2.50	±1.91	±2.65	±2.86	±1.44	±1.29	±2.49

*The bold values mean the best performance results.*

**TABLE 4:** Classification results of the comparison algorithms in HC and MCI dataset.

	Accuracy	Sensitivity	Specificity	Precision	F-measure	G-mean	Jaccard
SPCA	86.84	**98.76**	73.68	79.17	91.01	90.70	84.19
	±3.20	±3.13	±3.36	±3.56	±2.75	±1.75	±3.09
LRR	84.21	84.21	84.21	84.21	86.23	87.15	76.85
	±2.64	±3.22	±2.36	±3.15	±3.05	±3.00	±2.57
LRDLSR	88.60	89.47	87.72	88.12	90.46	91.93	84.32
	±2.18	±3.20	±2.97	±2.64	±3.08	±3.03	±3.06
JSLC	89.47	98.32	78.95	82.61	92.04	92.46	86.41
	±2.77	±3.01	±3.38	±2.17	±3.05	±1.58	±3.03
NRLRL	90.79	92.11	89.47	90.08	94.29	94.51	89.47
	±2.68	±3.49	±1.69	±3.00	±2.39	±2.71	±1.83
Our algorithm	**93.42**	94.74	**92.11**	**92.46**	**95.43**	**95.48**	**91.40**
	±2.39	±1.93	±2.25	±1.69	±2.12	±2.06	±2.04

*The bold values mean the best performance results.*

(1) Alzheimer’s disease is a population suffering from AD, which has shown clinical symptoms. The EEG signal differentiation between AD and healthy people is the most significant, and the difference between EEG features is more obvious. Therefore, the classification performance in the dataset of AD and HC is high. Although the symptoms of MCI are not as significant as those of AD, there is a certain probability of AD. The difference between the EEG features and those of healthy people is also significant, and the difference between EEG features is also obvious, so the classification performance in the dataset of MCI and HC is also high. In addition, AD and MCI are mixed into one class in the third dataset of HC and (AD+MCI), which is significantly distinguishable from healthy EEG signals. Therefore, its classification performance is expectable. The classification accuracy of DSLRR algorithm in AD and HC is 97.74%. The classification accuracy of the DSLRR algorithm in MCI & and HC is 95.61%. The classification accuracy of the DSLRR algorithm in HC and MCI+AD is 98.42%. The classification accuracy of these three datasets is above 97.26%. The experimental results illustrate that DSLRR can better identify MCI and AD from HC.
(2) Compared with the first three datasets, the difference between EEG features between MCI and AD is relatively low. Therefore, the classification performance of each algorithm decreases to a certain extent in the MCI & AD dataset. However, we can see that the DSLRR algorithm still achieves the best values of accuracy, F-measure, G-mean, and Jaccard. On the one hand, through the joint learning of subspace and low-rank representation, the DSLRR algorithm can learn the robust and discriminant projection subspace. On the other hand, by making full use of Laplace manifold and LSR technologies, the DSLRR algorithm can exploit the structure knowledge and manifold structure information of EEG signals. Furthermore, the sum of the columns of each low-rank coefficient matrix **L** of 1 has a positive effect on the classification.
(3) The LRR algorithm can describe the correlation of data, and the coefficient matrix is low rank. However, this algorithm doesn’t consider the local structural characteristics of the data, and often cannot effectively exploit the discriminant information in the data. In this case, the LRR algorithm is not directly applicable to the EEG classification for AD recognition. The JSLC algorithm achieves good results in four datasets. JSLC is a low-rank representation model based on dictionary learning, which integrates discriminant information of samples into dictionary learning, and can also eliminate the influence of noise information on the classification model. This result shows that joint learning of low-rank representation and subspace learning is an effective means to solve EEG classification. The NRLRL algorithm conducts low-rank learning in the original data space. Its classification performance is lower than DSLRR in four datasets, which further shows that more data dimensions may not improve model performance. Due to the redundant information and noise in EEG data, it is effective to obtain the compact and discriminant feature representation through subspace learning and low-rank representation.

### Ablation Experiment

**FIGURE 2:**
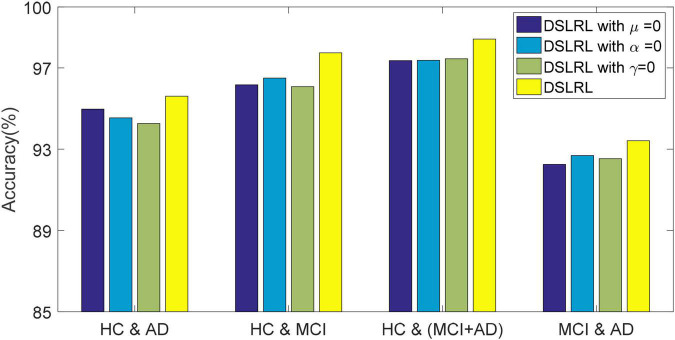
Classification accuracy of DSLRR with ablation experiment in four EEG datasets.

**FIGURE 3:**
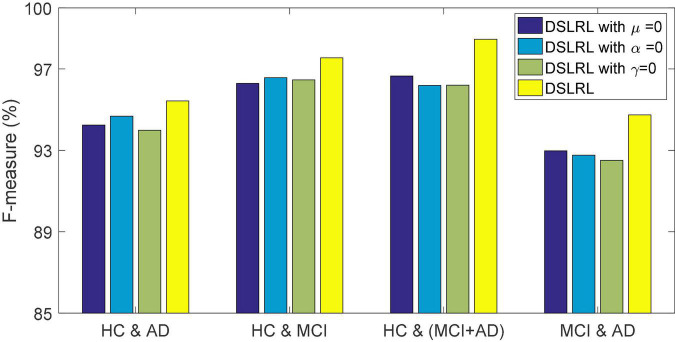
F-measure of DSLRR with ablation experiment in four EEG datasets.

**FIGURE 4:**
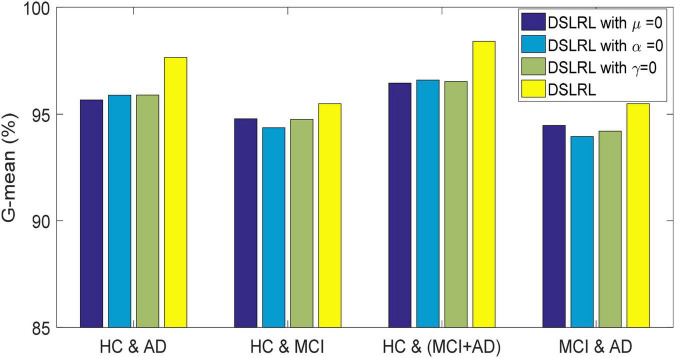
G-mean of DSLRR with ablation experiment in four EEG datasets.

The DSLRR algorithm integrates discriminant margin term, global structure term, and LSR term on the basis of the LRR algorithm. To verify the role of these terms, we performed ablation experiments on four EEG datasets. For discriminant margin term, its purpose is to use supervised information to establish graph embedding, to improve the distinguishing ability of the model. To verify its effect, we remove this item from Eq. (11), that is, set the parameter μ = 0. For global structure terms, their purpose is to preserve the structure information of data in subspace. To verify its effect, we remove this item from Eq. (11) by setting the parameter α = 0. For the LSR term, its purpose is to use the least square constraint to utilize the discriminant information in the data. Similarly, to verify its effect, we remove this item from Eq. (11), that is, set the parameter γ = 0. The classification accuracy, F-measure, and G-means of DSLRR with an ablation experiment in four EEG datasets are shown in Figures 2–4, respectively. From the results in Figure 2, we can see that if any one of three terms is removed from Eq. (11), the classification accuracy in the four EEG datasets has decreased to varying degrees. This is because each term has a corresponding contribution to the EEG classification task, which also illustrates the necessity of the coexistence of these three terms from another perspective. The results in Figures 3, 4 show that this conclusion is well verified. Therefore, the lack of any term will degrade the performance of the DSLRR algorithm.

### Parameter Analysis

**FIGURE 5:**
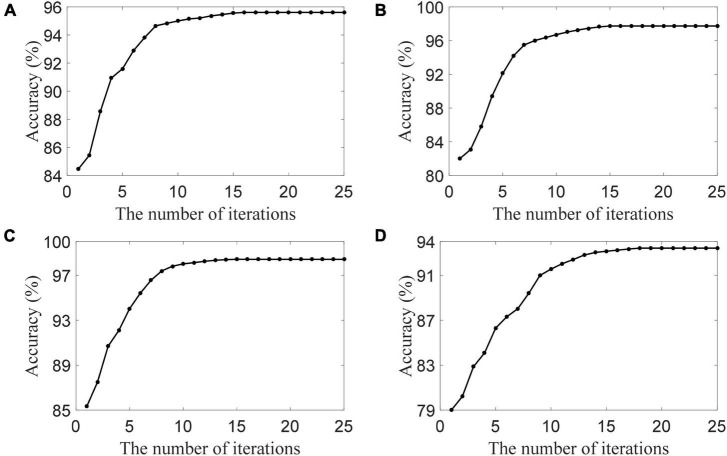
The convergence of the DSLRR algorithm in four datasets, **(A)** HC and AD, **(B)** HC and MCI, **(C)** HC and (MCI+AD), and **(D)** MCI and AD.

To show the convergence of the DSLRR algorithm, we plot its convergence curve in Figure 5. As shown in Figure 5, the DSLRR algorithm converges quickly in several iterations across four EEG datasets. The results show that the DSLRR algorithm is acceptable in the running time, which shows that the DSLRR algorithm has high practical worthiness.

**FIGURE 6:**
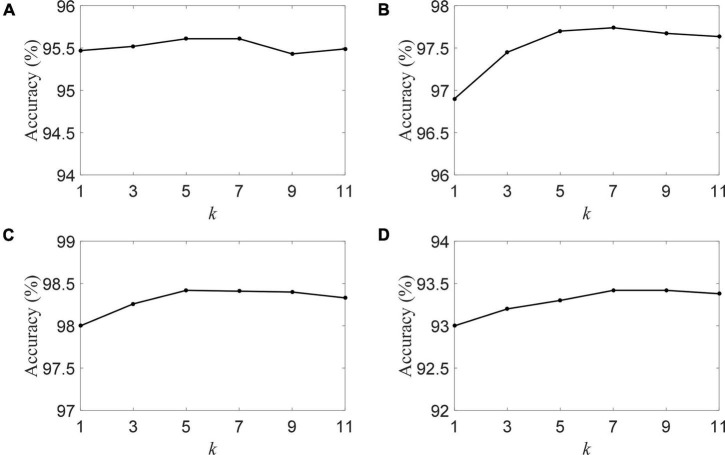
The accuracy of the DSLRR algorithm with different *k* in four datasets, **(A)** HC and AD, **(B)** HC and MCI, **(C)** HC and (MCI+AD), and **(D)** MCI and AD.

We plot the classification accuracy of the DSLRR algorithm with different *k*-nearest neighbors in Figure 6. Figure 6 visually shows that the classification is mildly sensitive to *k*. The DSLRR algorithm can achieve good classification accuracy when the parameter *k* is in the range of [5, 7, 9]. When *k* is <5 or *k* is greater than 9, the classification accuracy is slightly lower. Therefore, we can fix *k* = 7 in the experiment.

## Conclusion

With the emergence of global aging, the prediction and diagnosis of AD have attracted extensive attention. In recent years, EEG technology has been developed and has become an important means to detect abnormal brain activity in patients with AD. To realize the early diagnosis of AD, we propose the DSLRR learning algorithm. The DSLRR algorithm inherits the advantages of low-rank representation, removes redundant information and noise, and improves the discriminant ability of low-rank representation through graph discriminant embedding. Meanwhile, based on subspace learning, the DSLRR algorithm introduces LSR and global structure preserving constraints to further improve the discriminative ability of the model. Extensive experimental results on real EEG data verify the effectiveness of the DSLRR algorithm.

In the future, we will continue to explore our work in the following aspects. First, the DSLRR algorithm is essentially a linear learning method. The brain is a nonlinear system with the ability of self-adaptation and self-regulation. Under some internal or external stimuli, the regulation and application functions of biological tissue will inevitably affect the electrophysiological signals, so that neurons have chaotic discharge phenomena, which present nonlinear characteristics. This makes the DSLRR algorithm unable to exert its performance in complex EEG data. To this end, we consider introducing a nonlinear learning model to improve the stability and accuracy of the DSLRR algorithm, so that it can be better suitable for various complex application scenarios. Second, the DSLRR algorithm is suitable for EEG classification using single-feature information. At present, the technologies of feature processing and feature exaction are more mature, and the obtained feature information is correspondingly more diverse. In the next stage, we will extend the proposed algorithm to multi-feature scenarios to form a richer AD recognition system. Third, with the popularization of EEG acquisition equipment, using the existing labeled samples to analyze the unlabeled samples in multiple domains is a difficult problem in EEG-based AD recognition. We will use transfer learning technology to extend our algorithm in the future, to further enhance the generalization of the algorithm.

## Data Availability Statement

Publicly available datasets were analyzed in this study. The EEG dataset analyzed in this study can be found in: https://github.com/tsyoshihara/Alzheimer-s-Classification-EEG.

## Author Contributions

TT, XG, and JX conceived and developed the model and wrote the manuscript. HL and GZ ran the experiment and analyzed the results. All authors read, edited, and approved the manuscript.

## Conflict of Interest

The authors declare that the research was conducted in the absence of any commercial or financial relationships that could be construed as a potential conflict of interest.

## Publisher’s Note

All claims expressed in this article are solely those of the authors and do not necessarily represent those of their affiliated organizations, or those of the publisher, the editors and the reviewers. Any product that may be evaluated in this article, or claim that may be made by its manufacturer, is not guaranteed or endorsed by the publisher.
